# Decomposition of Reaching Movements Enables Detection and Measurement of Ataxia

**DOI:** 10.1007/s12311-021-01247-6

**Published:** 2021-03-02

**Authors:** Brandon Oubre, Jean-Francois Daneault, Kallie Whritenour, Nergis C. Khan, Christopher D. Stephen, Jeremy D. Schmahmann, Sunghoon Ivan Lee, Anoopum S. Gupta

**Affiliations:** 1 College of Information and Computer Sciences, University of Massachusetts Amherst, 140 Governors Dr, Amherst, MA USA; 2 Department of Rehabilitation and Movement Sciences, Rutgers University, 65 Bergen St, Newark, NJ USA; 3 Department of Neurology, Massachusetts General Hospital, Harvard Medical School, 100 Cambridge St, Boston, MA USA; 4 Ataxia Center, Department of Neurology, Massachusetts General Hospital, Harvard Medical School, 100 Cambridge St, Boston, MA USA; 5 Movement Disorders Unit, Department of Neurology, Massachusetts General Hospital, Harvard Medical School, 100 Cambridge St, Boston, MA USA

**Keywords:** Movement decomposition, Ataxia, Brief Ataxia Rating Scale, Machine learning, Wearable electronic devices

## Abstract

Technologies that enable frequent, objective, and precise measurement of ataxia severity would benefit clinical trials by lowering participation barriers and improving the ability to measure disease state and change. We hypothesized that analyzing characteristics of sub-second movement profiles obtained during a reaching task would be useful for objectively quantifying motor characteristics of ataxia. Participants with ataxia (*N*=88), participants with parkinsonism (*N*=44), and healthy controls (*N*=34) performed a computer tablet version of the finger-to-nose test while wearing inertial sensors on their wrists. Data features designed to capture signs of ataxia were extracted from participants’ decomposed wrist velocity time-series. A machine learning regression model was trained to estimate overall ataxia severity, as measured by the Brief Ataxia Rating Scale (BARS). Classification models were trained to distinguish between ataxia participants and controls and between ataxia and parkinsonism phenotypes. Movement decomposition revealed expected and novel characteristics of the ataxia phenotype. The distance, speed, duration, morphology, and temporal relationships of decomposed movements exhibited strong relationships with disease severity. The regression model estimated BARS with a root mean square error of 3.6 points, *r*^2^ = 0.69, and moderate-to-excellent reliability. Classification models distinguished between ataxia participants and controls and ataxia and parkinsonism phenotypes with areas under the receiver-operating curve of 0.96 and 0.89, respectively. Movement decomposition captures core features of ataxia and may be useful for objective, precise, and frequent assessment of ataxia in home and clinic environments.

## Introduction

Cerebellar ataxia is a neurologic phenotype caused by a heterogeneous set of underlying diseases that affect the function of the cerebellum [1]. Hereditary cerebellar ataxias (e.g., spinocerebellar ataxias, Friedreich’s ataxia, and ataxia-telangiectasia) occur at a rate of approximately 6 cases per 100,000 individuals [2]. Hereditary ataxias are generally progressive conditions, resulting in functional impairments, limited autonomy, and reduced quality of life over time [3, 4].

Quantification of ataxia severity is important for tracking disease progression and assessing the efficacy of potential treatments [5]. Clinical severity is currently assessed using clinician-performed rating scales [1], such as the Brief Ataxia Rating Scale (BARS) [6] and the Scale for the Assessment and Rating of Ataxia (SARA) [7]. Because administering existing clinical scales requires in-person motor examination by trained specialists, frequent assessment of severity can be logistically difficult. Furthermore, access to specialists—and thus assessment—is often limited in rural areas, underserved communities, and in populations with reduced mobility [8, 9]. The precision and inter-rater reliability of existing scales is also limited by their reliance on subjective, visual assessments that are influenced by each assessor’s clinical experience. Technologies that enable frequent, remote, and objective assessment of severity would, therefore, benefit clinical trials by lowering participation barriers and improving the ability to more sensitively and objectively measure disease state and progression. Towards this end, prior studies estimated ataxia severity using data collected from various sensing modalities including cameras [10], wearable sensors [11], instrumented spoons [12], and computer mice [13].

Miranda et al. recently demonstrated that complex three-dimensional (3D) hand movements can be viewed as a concatenation of short 1D submovements with zero initial and terminal velocities—which are referred to as *movement elements* [14]. The durations of these movement elements range from a few milliseconds to a couple of seconds, with an average of about 500 ms [15]. Moreover, movement elements’ velocities resemble consistent, bell-shaped curves in neurologically healthy individuals resulting from smooth acceleration and deceleration [14]. Because less smooth, corrective, and segmented movements are core features of ataxia [16], we hypothesized that movement elements extracted from ataxic movements should exhibit more varied velocity profiles and temporal patterns reflecting increased and potentially disordered segmentation. Furthermore, the scoring criteria of BARS and the International Cooperative Ataxia Rating Scale (ICARS) [17] suggest that segmentation of movement increases with disease severity on the finger-to-nose test (FNT). Prior work has also demonstrated that information extracted from the FNT is related to the presence of ataxia and its severity [10, 18, 19].

To determine if movement elements extracted from reaching movements encode ataxia severity, participants were equipped with wrist-worn inertial measurement units (IMUs) as they performed the FNT. Movement elements were extracted from participants’ accelerometry data and summarized by a set of features engineered to capture ataxia-related changes. Machine learning models were trained using these features to estimate overall ataxia severity and to distinguish between ataxia and healthy controls and between ataxia and parkinsonism.

## Methods

### Participant Selection

Eighty-eight participants with clinically diagnosed ataxias, 44 with clinically diagnosed parkinsonism, and 34 neurologically healthy control participants were recruited from the Massachusetts General Hospital and in collaboration with the Ataxia-Telangiectasia Children’s Project (see Table 1 for participant demographics). Ataxia diagnoses included a diverse set of underlying conditions. Participants with a clinical diagnosis of possible or probable multiple system atrophy were categorized as ataxia-dominant or parkinsonism-dominant based on their clinical phenotypes. One individual with a clinical diagnosis of progressive supranuclear palsy had predominant cerebellar ataxia and was categorized under the ataxia phenotype. Four participants were diagnosed with presumed autoimmune-related ataxia based in part on their response to immunosuppressant therapy. Inclusion criteria were that participants were 1) between 2 and 90 years old; 2) were able to perform the instrumented FNT; and 3) had a clinical diagnosis of ataxia or parkinsonism, or were neurologically healthy. Fifteen individuals with ataxia, five with parkinsonism, and two controls participated in the experiment multiple times during separate visits.

**Table 1:** Participant demographics

	Control	Ataxia	Parkinsonism
***N***	34	88 (total) 4 SCA-1, 2 SCA-2, 11 SCA-3, 7 SCA-6, 10 other SCA, 7 A-T, 3 FA, 7 MSA-C, 1 PSP-C, 3 HSP, 4 AIA, 1 BD, 1 HE, 2 EA, 2 ARCA-1, 1 ARCA-3, 1 CH, 3 DN, 2 SA, 1 FXTAS, 1 GHS, 1 LCHND, 1 SRA, 3 TA, 2 SAOA, 5 SAOAN, 2 ADCA	44 (total) 42 idiopathic PD, 1 MSA-P, 1 PSP
Age	21–86 (M ± SD 39.0 ± 18.2)	5–78 (M ± SD 54.4 ± 18.5)	45–85 (M ± SD 67.5 ± 8.2)
Sex	13 male, 21 female	47 male, 41 female	31 male, 13 female
Handedness	33 right, 1 left	78 right, 9 left, 1 ambidextrous	41 right, 3 left
Disease Severity (total clinical score on BARS [ataxia] or UPDRS [parkinsonism])		0–24 (M ± SD 10.5 ± 5.4)	3–51 (M ± SD 16.3 ± 9.1)

*M* mean; *SD* standard deviation; *UPDRS* Unified Parkinson’s Disease Rating Scale (Part III Motor Examination); *BARS* Brief Ataxia Rating Scale; *SCA* spinocerebellar ataxia; *A-T* Ataxia-Telangiectasia; *FA* Friedreich’s Ataxia; *MSA-C* multiple system atrophy, cerebellar type; *MSA-P* multiple system atrophy, parkinsonian type; *PSP-C* progressive supranuclear palsy, cerebellar-dominant; *PSP* progressive supranuclear palsy; *HSP* hereditary spastic paraplegia; *AIA* autoimmune-related ataxia with undefined cause; *BD* Behcet’s Disease; *HE* Hashimoto’s Encephalopathy; *EA* episodic ataxia; *ARCA* autosomal recessive cerebellar ataxia; *CH* cerebellar hypoplasia; *DN* Downbeat Nystagmus with mild ataxia; *SA* sensory ataxia; *FXTAS* Fragile X-Associated Tremor/Ataxia Syndrome; *GHS* Gordon Holmes’ Syndrome; *LCHND* LCH-related neurodegeneration; *SRA* stroke-related ataxia; *TA* transient ataxia, later resolved; *SAOA* sporadic adult-onset ataxia; *SAOAN* sporadic adult-onset ataxia with neuropathy; *ADCA* autosomal dominant cerebellar ataxia with unidentified genetic cause; *PD* Parkinson’s Disease

### Data Collection

**Fig. 1:**
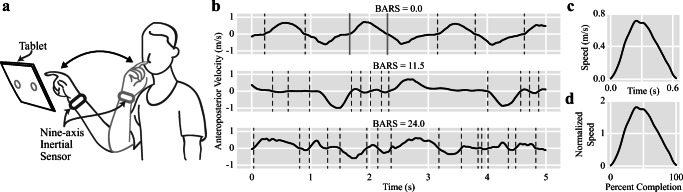
a) An illustration of a participant performing the instrumented finger-to-nose test. b) Five seconds of the anteroposterior velocity time-series computed from wrist-worn inertial sensor data. The time-series of three participants with different Brief Ataxia Rating Scale (BARS) scores are shown. Movement elements are obtained by segmenting the time-series at the velocity zero-crossings, which are denoted by dashed vertical lines. Movement elements are also independently obtained from both the mediolateral and rostrocaudal velocity time-series. c) The movement element denoted by the solid gray vertical lines. d) The movement element denoted by the solid gray lines after spatial and temporal normalization. The shape is the same, but the velocity has been divided by its mean and resampled

Each participant’s overall motor impairment severity was assessed using BARS (range 0–30, in half-point increments) for the ataxia group, and the Unified Parkinson’s Disease Rating Scale (UPDRS) Part III Motor Examination [20] (range 0–108) for the parkinsonism group. Higher scores indicate greater motor disease severity for both scales. BARS, which is based on the Modified ICARS and correlated to both SARA and ICARS, was used as the assessment tool for participants with ataxia because of its brevity and because its scoring criteria for the FNT emphasize segmentation of movement [21]. Participants were equipped with nine-axis IMUs (Opal, APDM Wearable Technologies) on both wrists and seated upright with their feet firmly on the floor in front of a 12.9 in. tablet device (iPad Pro, Apple Inc.) positioned at approximately 90% arm's reach away in the midline of the body (i.e., in the frontal plane) and at the participant’s eye level (Fig. 1a). The tablet displayed a circular target with a 1.5-cm diameter that alternated between the left and right sides of the screen every 10 s. The position of the tablet and size of the target were chosen to emulate the physician-performed FNT [22], and the 10-s interval was chosen to allow participants to perform multiple reach and return sequences for each target position. Participants performed a continuous, 40-s FNT task with each hand (i.e., a total of 80 s). During each task, participants repeatedly moved their finger between their nose and the circular target as quickly and accurately as possible.

### Pre-processing of Inertial Data

Inertial data were sampled at 128 Hz. Gravity was removed from acceleration time-series by subtracting the mean and a sixth order low-pass Butterworth filter with a cutoff frequency of 20 Hz was used to remove high frequency noise from non-human sources [23]. Filtered acceleration time-series were trapezoidally integrated to obtain velocity time-series. Because the reaching target changed positions every 10 s, a sixth order band-pass Butterworth filter with cutoff frequencies of 0.1 Hz and 20 Hz was used to remove integration drift and high frequency noise from velocity time-series [23].

To apply movement decomposition [14], velocity time-series must be oriented with the body’s anatomical axes. Because participants sat upright and repeatedly moved a finger between their nose and a tablet positioned directly in front of them, the rostrocaudal axis was aligned with gravity and the anteroposterior axis was assumed to be in the direction of greatest variance of the velocity time-series identified using Principal Component Analysis [24] (PCA). Each axis of the body-oriented 3D velocity time-series was independently segmented into movement elements at its zero-crossings (i.e., when the velocity is zero) (Fig. 1b) [15]. As in prior work, movement elements smaller than 1 mm or shorter than 5 ms—accounting for 3.0% of the combined total duration of all movement elements—were regarded as potential sensor noise and excluded from further analysis [15].

### Feature Extraction

Fifty-three features hypothesized to be relevant to ataxia severity were extracted from each participant’s movement elements. Movement elements from both hands were pooled together for feature calculations. It was hypothesized that less-impaired participants would generate large, consistent movement elements corresponding to relatively smooth reaching motions between the nose and tablet. For example, prior work showed that healthy participants performing a 3D reaching movement towards a target (i.e., reaching for a can of soda) generated velocity profiles dominated by a large, primary movement element in each of the three axes [14]. In contrast, it was hypothesized that more severe participants would perform increasingly segmented, oscillatory, and irregular movements corresponding to smaller, more variable movement elements. In particular, it was hypothesized that dysmetria would induce the generation of many small movement elements with alternating directions reflecting corrective movements.

To capture the size and speed of movement elements, their time durations, distances, and mean speeds were computed. Logarithms of distances and mean speeds were used to emphasize the differences between smaller movement elements and because prior work suggested that the logarithms of the two variables would be approximately linearly related [14]. Each of these attributes were aggregated for each participant using the mean, standard deviation, minimum, maximum, range, interquartile range, median, and tenth and ninetieth percentiles. In neurologically healthy participants, the distance (*D*) and mean speed ($\overline{v}$) of movement elements are related by the two-thirds power law [14], $\overline{v}\propto {D}^{\alpha }$ where *α* = 2/3. A decrease in this scaling exponent (α) indicates that slower velocities are generated to achieve a particular movement distance. Because it was expected that ataxia patients would generate slower movements [16], α was computed as a feature by fitting a least-squares linear regression between the logarithms of the distances and logarithms of the mean speeds for each participant and extracting the slope of the regression line.

Temporal relationships were captured by analyzing changes in distance and direction across consecutive movement elements. The probability density of transitions between consecutive movement elements was estimated using a normalized 2D histogram of the signed distances (i.e., signed distances of the prior vs. subsequent movement elements). In other words, each bin of the 2D histogram represented the probability of two consecutive movement elements having a particular distance and direction. To reduce the number of data features required by our model, only histogram bins corresponding to transitions between small movement elements were included as data features because it was hypothesized that transitions between small movements would be more common in ataxic movements as a result of dysmetria (Fig. 3). The ratios of consecutive unsigned movement element distances were computed to understand how movement element distances changed across consecutive movements when considering movements with both small and large distances. The mean, standard deviation, minimum, maximum, range, interquartile range, median, and tenth and ninetieth percentiles of the ratios were extracted as data features.

To capture differences in the morphology of movement elements’ velocity profiles—which relates to the smoothness and pattern of acceleration and deceleration—movement elements were spatially normalized by dividing them by their mean velocities and then temporally normalized by resampling them to sixty samples (Fig. 1c and d), which was chosen based on the median duration of the extracted movement elements [14, 15]. PCA was used to summarize the 60D normalized movement elements in a Leave-One-Subject-Out manner. Statistical aggregations (i.e., the mean, standard deviation, minimum, maximum, range, interquartile range, median, and tenth and ninetieth percentiles) of the first two principal components representing each normalized movement element were extracted as features.

### Estimation of Clinical Scores

To estimate participants’ total BARS scores, a regression model (Gaussian Process Regression [25] with a Radial Basis Function kernel) was trained and evaluated using Leave-One-Subject-Out Cross Validation. Pediatric participants were excluded from the training data to reduce the possibility of the model learning trends corresponding to immature motor patterns. Features were scaled such that the training set had a fixed range [26] and clinician-assessed scores were normalized such that the training data had zero mean and unit variance. A similar model was also trained to estimate participants’ summed upper-limb BARS scores (range 0–8). Total BARS was used as the primary label given that the total score has increased granularity, may be more robust to error as it integrates information from several domains, and to support the goal of identifying arm movement properties that relate to overall disease severity.

Estimation performance was evaluated using *r*^2^ and the root mean square error (RMSE) of the model-estimated and clinically-assessed scores. The Pearson correlation of the estimation errors to participant ages was computed to determine if the model was biased by age. Reliability was evaluated using participants who received multiple assessments during several visits. Each visit was separated by several months (283.4 ± 106.1 days; range 126–433 days). Pearson correlation and a Welch’s *t*-test were used to determine if changes in clinician-assessed and estimated BARS were in agreement for participants with multiple assessments. A single rater, consistency, two-way mixed-effects model was used to calculate the intraclass correlation (ICC(3, 1)) and 95% confidence interval (CI) of the repeated model estimates.

To further understand the contribution of dominant and nondominant hand data in estimating clinical severity and to further assess model consistency, BARS scores were estimated based on data features calculated from each hand, separately. (i.e., the model training process was unaltered and additional hand-specific estimates were computed.) Pairwise correlations were calculated between dominant-hand-only estimates, nondominant-hand-only estimates, and the original estimates based on data from both hands.

### Ataxia Classification

To evaluate the specificity of the extracted features to ataxia, classification models (Gaussian Process Classifier [25] with a Radial Basis Function kernel) were trained to distinguish between ataxia participants and healthy controls and between individuals with ataxia and parkinsonism. The classification models were trained and evaluated using the same pipeline as the regression model and performance was evaluated using the area under the receiver-operating curve (AUC) and its 95% CI. Because the populations were not age-matched, AUCs for participants in each decade of life were computed and Pearson correlation was used to determine if a relationship between age and model error existed. Additional classification models were also trained using only participants between 18 and 45 years old and using only participants at least 45 years old to mitigate the possibility that the classifiers were leveraging age-related differences in motor performance [27].

### Analyzing Morphological Changes During Task Performance

Based on the results of the analysis of movement element morphologies, we investigated if a motor optimization process was occurring during the performance of the FNT. Optimization of normalized movement elements has been observed in healthy participants as they become more proficient at a task [28]. More specifically, the morphology of normalized movement elements converges to the theoretical model proposed by Hoff for 2D point-to-point movements [29]. To determine if optimization occurred during the performance of the FNT, three metrics were computed from the first 20 s and the last 20 s of each participant’s 40-s FNT time-series for each hand: 1) the standard deviation of the first principal component representing each movement element’s morphology, 2) the standard deviation of the second principal component, and 3) the coefficient of determination (*r*^2^) between each normalized movement element and the theoretical model. Decreases in the standard deviations of the principal components indicated more consistent morphologies in the second half of the test. Increases in *r*^2^ indicated morphologies with greater similarities to the theoretical model in the second half of the test. Significant changes were identified using a one sample *t*-test with a theoretical mean of zero (i.e., no change).

### Additional Statistical Analyses

A significance level of *p* < 0.05 was used for all tests. Welch’s ANOVA was used to determine if significant differences existed between the number of movement elements extracted from each participant in the three populations. Pearson correlation and Welch’s *t*-tests were used to measure the strength of relationships between individual features and BARS, and to determine if features were significantly different for healthy and ataxia participants, respectively. Pearson correlation was used to determine the strength of relationships between features and age.

To test for significant differences between feature values, participants were divided into four groups based on their total BARS severity. The healthy group consisted of controls (*N*=34) and three ataxia groups were determined by equally partitioning the range of BARS scores present in the collected data (total BARS groups 0–8, *N*=30; 8.5–16, *N*=44; and 16.5–24, *N*=15). One participant’s data were included in two groups because their severity increased between subsequent visits. Significant differences were determined using Welch’s ANOVA and Games-Howell post-hoc tests.

## Results

The number of movement elements extracted from each participant’s accelerometry data was 456.1 ± 83.9 for the healthy population, 448.6 ± 96.0 for the ataxia population, and 411.0 ± 108.9 for the parkinsonism population. The population means were not significantly different (*F* = 2.70; *p* = 0.07).

### Relationships of Features to the Clinical Severity

**Fig. 2:**
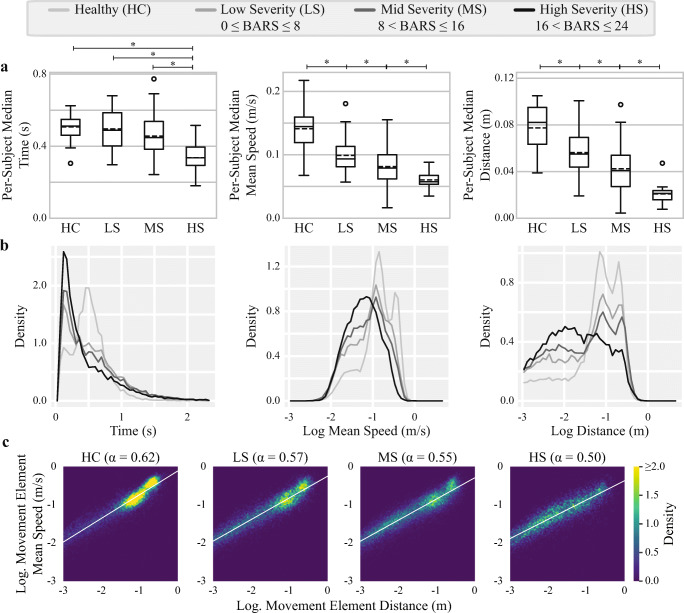
a) Box plots illustrating the per-participant duration, mean speed, and distance of movement elements for four groups of participants. Circles denote outliers and dashed lines denote the mean of each group. Participants were assigned to each group based on their Brief Ataxia Rating Scale (BARS) scores. Horizontal whiskers and asterisks along the top of each plot denote significantly different means, determined using Welch’s ANOVA and Games-Howell post-hoc tests for *p* < 0.05. All groups are significantly different for the mean speed and distance. b) Probability density estimates for all movement elements in each of the severity groups, calculated using histograms with uniformly spaced bins. c) Identification of the power-law scaling exponent (*α*) between movement element distance and mean speed for each severity group. The white line is the least squares linear regression

Movements were segmented into shorter, smaller, and slower segments in individuals with more severe ataxia (Fig. 2a). Significant differences were identified between all groups for mean speed and distance, and between the high severity group and other groups for the duration. The probability densities of movement element duration, mean speed, and distance appear to change as a function of disease severity, gradually diverge from the shape of the healthy distribution as severity increases, and are distinctly different for the high severity groups (Fig. 2b). In particular, the density of the traveled distances in the region corresponding to the full FNT reaching distance decreases with severity. The power law relationship between the movement element distances and mean speeds also showed significant differences between the four severity groups, with lower mean scaling exponents (α) corresponding to higher severity (Fig. 2c). The decrease in *α* indicates that more severe participants performed slower movements for a given distance than less severe participants.

**Fig. 3:**
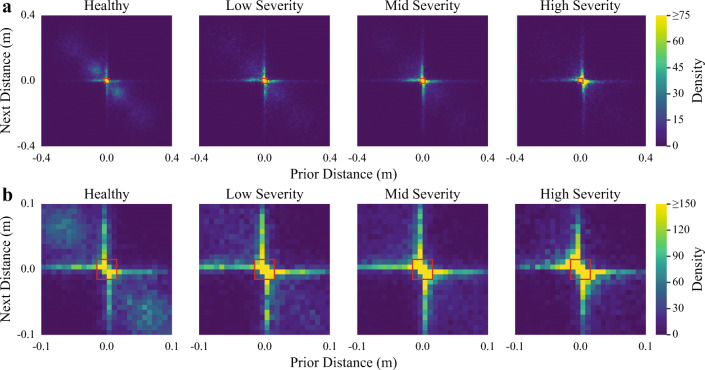
a) A comparison of the distances and directions of consecutive movement elements for each of the four severity groups detailed in the methods. Less-severe groups are more likely to perform large, alternating movements as shown by the density of the diagonal trend. More-severe groups are more likely to perform very small movements as shown by the density of the center region. The red box at the center of the histograms denotes the region used to extract data features for the machine learning models. b) The same comparison, using a smaller range of movement distances to more clearly demonstrate differences between the four severity groups. Densities have been recalculated with respect to the smaller range of the histogram

Features capturing temporal changes between consecutive movement elements also demonstrated strong relationships with disease severity (Fig. 3). A strong diagonal trend, corresponding to the large alternating movements required by the task itself, is present in the distribution for the healthy group but fades in the increasingly severe groups, which instead show a greater density of very small consecutive movements.

**Fig. 4:**
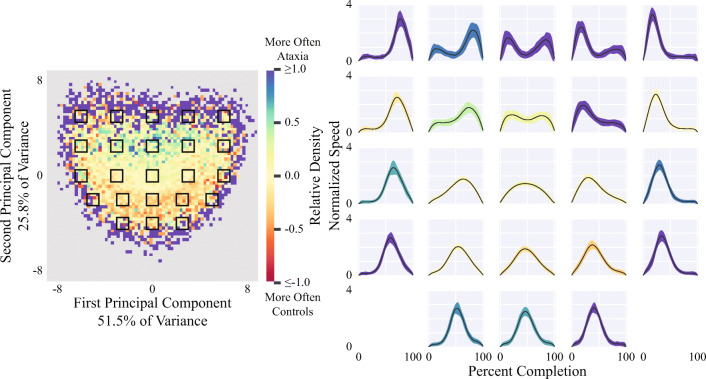
Movement element shapes in ataxia and control participants. The left plot shows the distribution of spatially and temporally normalized movement elements based on their first two principal components, obtained from a principal component analysis of all ataxia participants and healthy controls. The color corresponds to the relative density of movement elements of ataxia participants and healthy controls. The relative density is calculated as the logarithm of the ratio of movement element densities. A value of 1.0 indicates that movement elements from ataxia participants are ten times more likely to occur and a value of -1.0 indicates that movement elements from healthy controls are ten times more likely to occur. The black boxes in the left plot correspond to the grid of plots on the right. Each plot on the right shows the mean (black line) and standard deviation (shading) of normalized movement elements within the corresponding black box. The color of the shading represents the mean value of the bins within the corresponding box

**Fig. 5:**
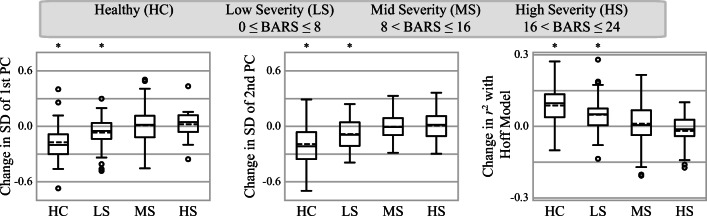
Box plots illustrating changes in the morphology of movement elements between the first and second halves of the finger-to-nose test for four groups of participants. Circles denote outliers and dashed lines denote the mean of each group. Participants were assigned to each group based on their Brief Ataxia Rating Scale (BARS) scores. A decrease in standard deviation (SD) of each principal component (PC) represents more consistent movement element morphology in the second half of the test. An increase in *r*^2^ with the Hoff model indicates more-optimized movement elements in the second half of the test. Asterisks along the top of each plot denote a significant change between the first and second halves of the test for the group, determined using a one sample *t*-test for *p* < 0.05

Movement elements demonstrated morphological trends related to severity (Fig. 4). The first principal component captured both the sharpness of movement elements’ peaks, which corresponds to the smoothness of acceleration, as well as the possible occurrences of multiple peaks. The second principal component captured the skewness of movement elements, which represents asymmetric acceleration. For example, a left-skewed movement element indicates an initially jerky motion that gradually slows. Ataxia participants were more likely to have a larger range of movement element morphologies with respect to both principal components, indicating that ataxic movements had more varied and atypical velocity profiles. Furthermore, healthy controls and the low severity group (but not the more severe ataxia groups) demonstrated increased consistency in movement element morphologies and convergence towards the theoretically optimal model in the latter 20 s of each FNT task (Fig. 5).

Many individual features had strong correlations with BARS. In particular, the mean and median of movement element distances, the variance of the ratios of consecutive movement element distances, and the value of the power law scaling exponent (*α*) had strong Pearson correlations with BARS (i.e., |*r*| > 0.7; *p* < 0.01). These simple features together indicate that more severe participants generated smaller, slower movement elements with a higher chance of transitioning between movement elements of different sizes. The ten features with the highest correlation to clinical severity (Table 2) also exhibited significant differences between ataxia participants and healthy controls (*p* < 0.01).

**Table 2:** The ten data features with the strongest Pearson correlation coefficients (*r*) with respect to the clinician-assessed Brief Ataxia Rating Scale scores. All correlations have *p* < 0.01

*r*	Feature description
-0.73	Mean of movement element distances (in the log scale).
0.72	Interquartile range of the (log) ratios of consecutive movement element distances. Higher values correspond to less consistency in the distances traveled during consecutive submovements.
-0.72	The power-law scaling exponent (α) between movement element distances and mean speeds.
-0.71	Median of movement element (log) distances.
-0.69	Median of movement element (log) mean speeds.
-0.69	Mean of movement element (log) mean speeds.
0.65	Density of small consecutive movement elements with different sizes and the same direction (towards the tablet). Higher values likely correspond to small, more segmented movements during the reaching behavior.
-0.64	Ninetieth percentile of movement element (log) mean speeds.
0.64	Density of small consecutive movement elements with the same size and direction (towards the tablet). Higher values likely correspond to small, more segmented movements during the reaching behavior.
0.63	Standard deviation of the (log) ratios of consecutive movement element distances. (i.e., Higher values correspond to less consistency in the distances traveled during consecutive submovements

### Estimation of Clinical Scores

**Fig. 6:**
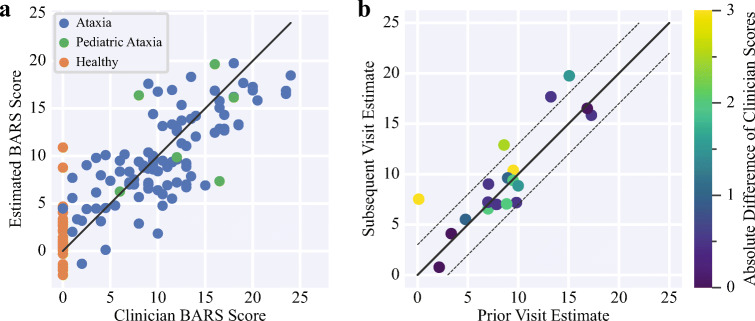
a) A scatter plot showing clinician-assessed Brief Ataxia Rating Scale (BARS) scores versus the scores estimated by the machine learning model. The solid black line indicates perfect model performance. b) A scatter plot demonstrating the reliability of the machine learning model by showing changes in the model’s estimates for participants with multiple assessments. The color indicates the absolute change in the clinician-assessed Brief Ataxia Rating Scale (BARS) score. The solid black line indicates that the same score was estimated for each visit and the dotted black lines indicate a difference of 3 BARS points between visits

The regression model was able to estimate BARS with an RMSE of 3.6 points and *r*^2^ = 0.69 (Fig. 6a). No significant correlation was observed between model error and participant age (*r* =  − 0.16; *p* = 0.05). Estimated BARS scores for healthy controls, excluding two outliers (in their third and sixth decades of life), had a mean of 1.4 and a range of −2.5–4.8. The two outliers had BARS estimates of 10.8 and 8.6; visual reexamination of the recorded tasks revealed that one outlier exhibited mild slowness and a mild intention tremor, and the other outlier performed the FNT faster than recommended and briefly switched hands in the middle of the task. The estimated scores of four participants notably increased between subsequent visits. There was no significant difference (*t* = 0.03; *p* = 0.97) between the changes in the clinician-assessed and model-estimated scores, indicating no bias in the model’s repeated measurements with respect to the clinicians’ measurements. The correlation between the changes in the clinician-assessed and changes in the model-estimated scores was 0.57, *p* = 0.01. For individuals with at least two assessments, ICC(3, 1) was 0.85 (*p* < 0.01; 95% CI of 0.64–0.94), which indicates moderate-to-excellent reliability [30] (Fig. 6b). BARS estimates obtained from features calculated using only nondominant-hand data were very strongly correlated with estimates obtained from features calculated using only dominant-hand data (*r* = 0.96; *p* < 0.01). Furthermore, estimates based on data from both hands (i.e., Fig. 6a) were very strongly correlated with both nondominant-hand-only estimates (*r* = 0.99; *p* < 0.01) and dominant-hand-only estimates (*r* = 0.99; *p* < 0.01).

The proposed model also estimated the summed upper-limb subscores of BARS with an RMSE of 0.9 points and *r*^2^ = 0.65. This similar performance was expected given the strong correlation between total BARS and the upper-limb subscores (*r* = 0.90, *p* < 0.01).

### Ataxia Classification

The AUC of the classification model for distinguishing between ataxia participants and healthy controls was 0.96 (95% CI of 0.94–0.99). No significant correlation was observed between age and per-decade AUC (range 0.93–1.00; *r* = 0.26, *p* = 0.62). The AUC of the control classifier was 0.93 (95% CI of 0.84–1.00) when only including participants between 18 and 45 years old (*N* = 42) and when only including participants at least 45 years old (*N* = 92) was 0.94 (95% CI of 0.90–0.99). The AUC of the classification model for distinguishing the ataxia and parkinsonism phenotypes was 0.89 (95% CI of 0.85–0.94). No significant correlation was observed between age and per-decade AUC (range 0.85–0.95; *r* = 0.44, *p* = 0.56). The AUC of the phenotype classifier was 0.90 (95% CI of 0.85–0.95) when only ataxia participants that were at least 45 years old were included (*N* = 131). The AUCs of the age-specific models suggest that performance was not dependent on age-related trends, although the ages within each population were not controlled. These results support that the extracted features can distinguish between ataxia and controls and between ataxia and parkinsonism with high accuracy.

## Discussion

Decomposing arm movements into discrete, typically sub-second movement elements is a novel and useful approach for representing and quantifying the ataxia phenotype. Characteristics of movement elements including their distance, speed, duration, morphology, and temporal relationships exhibited strong relationships with disease severity and were significantly different between ataxia participants and healthy controls. The performance of the models for distinguishing a diverse set of ataxia diagnoses from controls and parkinsonism indicate that movement elements capture characteristics relevant and specific to the ataxia phenotype.

### Ataxia Representation

Existing clinical scales involve semi-quantitative rating of clinical signs, such as movement segmentation [6] and intention tremor [7], to evaluate disease severity. Prior approaches using inertial data analyzed the position trajectory of the hand during the FNT [18]; analyzed the acceleration, velocity, and angle (including the frequency domains) of the FNT, dysdiadochokinesia test, and heel-shin test [11]; and analyzed the stability, timing, accuracy, and rhythmicity of tests corresponding to multiple motor domains [31]. The movement element-based approach demonstrated herein provides a complementary and natural lens through which the ataxia phenotype can be examined. Movement trajectory cohesiveness and segmentation is captured by the speed, duration, and distance of individual movement elements, which change as a function of disease severity (Fig. 2). Small corrective movements and dysmetria are captured by the temporal patterns of consecutive movement elements, i.e., with increasing disease severity, participants were more likely to transition between short distance movement elements. This finding is corroborated by similar works demonstrating the presence of directional changes and small corrective movements during performance of the FNT and other related reaching tasks [10, 13, 32].

Additionally, the power-law relationship between movement element distance and mean speed was strongly altered in individuals with ataxia (Fig. 2c). It is possible that decreased speed for a given distance is compensation by the motor system for reduced movement accuracy [28]. The distribution of normalized movement element morphologies also indicates that ataxia participants generate a broader range of velocity profiles (Fig. 4). Movement element profiles in ataxia were more sharply peaked, left or right-skewed, and had multiple peaks suggesting less smooth and less regular movement components during arm reaching. Investigation of these varied profiles may provide additional insight into how cerebellar disorders influence motor performance [14]. The results also suggest that healthy controls optimized aspects of their performance throughout the FNT and that this optimization process was impaired in individuals with more severe ataxia. Further study is needed to determine if and how these observed phenomena are related to impairments in motor learning [33]. One additional advantage of the movement element representation is that movement elements can be extracted from any goal-directed 3D movement, including natural arm behaviors, but also leg movements and gait [28]. Thus, movement element-based analysis may be able to capture dysmetria and segmented movements during walking or the heel-to-shin test and may capture ataxia from passive wearable sensor recordings during natural behavior.

### Severity Estimation

Technologies for precise and objective estimation of disease severity are needed to reduce the cost, duration, and sample size of clinical trials in ataxia. Measurements need to be interpretable and meaningful, reliable, correlated with gold standard assessments, and sensitive to disease change. The proposed regression model for estimating disease severity was based on a small number of interpretable and intuitive movement element features, and demonstrated strong agreement with the clinical ataxia rating scale. Furthermore, the data set used in this study is relatively large with respect to prior studies and includes a diverse range of diagnoses, which suggests that the presented results well-represent the phenotype and will generalize to other ataxia populations. The regression model demonstrated moderate-to-excellent reliability despite the elapsed time between subsequent measurements, and increased its estimation of the severity for four participants on the second assessment (Fig. 6b). This finding is promising and, while the change observed in certain participants may stem from noise or other factors, it may indicate that the movement element representation could be sensitive to disease progression in some individuals. Longitudinal study is needed to further investigate this possibility and compare the responsiveness of movement element features with other measures. Future work combining movement element-based severity estimation with low-burden assessments of other motor domains, such as speech and eye movements [6, 31], may enable more accurate, robust, and sensitive estimations. Similarly, given the highly accurate ataxia versus control classification performance, the approach used herein may be useful as a component of an ataxia screening tool, although evaluation in individuals with early-stage ataxia is needed.

Given the ubiquity and low cost of wearable sensors and computer tablets, we envision that the presented computer tablet variant of the FNT can be deployed in patients’ home settings to assess ataxia severity as part of observational and interventional research studies. Home-based assessment tools, such as this one, could reduce burden and extend accessibility of clinical research to rural and underserved communities. Furthermore, this assessment could allow for more frequent sampling of behavior, enabling reduced variability and increased precision of severity estimates.

## Limitations

Many of the presented data features will likely vary as a function of age/developmental stage and arm length (e.g., someone with a shorter arm will produce movement elements with smaller distances). Additional investigation of how the presented features vary with respect to these factors could further improve model performance and allow for an understanding of how movement elements differentially evolve with normal and abnormal motor development. Though this study analyzed the motion of the wrist to leverage the acceptability and accessibility of wrist-worn sensors, analyses of finger-tip motion may yield additional information. Although controls were screened for neurologic impairments, it is possible that subtle motor signs could have negatively impacted model estimates, as seen in one outlier described in the “Results” section. Though our results indicate that the extracted features are robust to age-related motor differences, comparison with an age-matched data set would further validate this finding. Further study is needed to separate the contributions of motion decomposition, intention tremor, and truncal ataxia on observed movement elements derived from the wrist sensor data. Additional longitudinal data is also necessary to determine if the motor optimization effects observed in this study diminish with repeat performances of the FNT.

## References

[1] Ashizawa T, Ataxia XG (2016). Continuum.

[2] Ruano L, Melo C, Silva MC, Coutinho P (2014). The global epidemiology of hereditary ataxia and spastic paraplegia: a systematic review of prevalence studies. Neuroepidemiology..

[3] López-Bastida J, Perestelo-Pérez L, Montón-Alvarez F, Serrano-Aguilar P (2008). Social economic costs and health-related quality of life in patients with degenerative cerebellar ataxia in Spain. Mov Disord.

[4] Wilson CL, Fahey MC, Corben LA, Collins VR, Churchyard AJ, Lamont PJ (2007). Quality of life in Friedreich ataxia: what clinical, social and demographic factors are important?. Eur J Neurol.

[5] Friedman LS, Farmer JM, Perlman S, Wilmot G, Gomez CM, Bushara KO (2010). Measuring the rate of progression in Friedreich ataxia: implications for clinical trial design. Mov Disord Wiley Online Library.

[6] Schmahmann JD, Gardner R, MacMore J, Vangel MG (2009). Development of a brief ataxia rating scale (BARS) based on a modified form of the ICARS. Mov Disord.

[7] Schmitz-Hübsch T, du Montcel ST, Baliko L, Berciano J, Boesch S, Depondt C (2006). Scale for the assessment and rating of ataxia: development of a new clinical scale. Neurology..

[8] Schuller KA, Vaughan B, Wright I (2017). Models of Care Delivery for Patients With Parkinson Disease Living in Rural Areas. Fam Commun Health.

[9] Saadi A, Himmelstein DU, Woolhandler S, Mejia NI (2017). Racial disparities in neurologic health care access and utilization in the United States. Neurology..

[10] Jaroensri R, Zhao A, Balakrishnan G, Lo D, Schmahmann JD, Durand F, Doshi-Velez F, Fackler J, Kale D, Ranganath R, Wallace B, Wiens J (2017). A Video-Based Method for Automatically Rating Ataxia. Proceedings of the 2nd Machine Learning for Healthcare Conference.

[11] Krishna R, Pathirana PN, Horne M, Power L, Szmulewicz DJ (2019). Quantitative assessment of cerebellar ataxia, through automated limb functional tests. J Neuroeng Rehabil.

[12] Nguyen KD, Corben LA, Pathirana PN, Horne MK, Delatycki MB, Szmulewicz DJ (2020). The Assessment of Upper Limb Functionality in Friedreich Ataxia via Self-Feeding Activity. IEEE Trans Neural Syst Rehabil Eng.

[13] Gajos KZ, Reinecke K, Donovan M, Stephen CD, Hung AY, Schmahmann JD (2020). Computer mouse use captures ataxia and parkinsonism, enabling accurate measurement and detection. Mov Disord.

[14] Miranda JGV, Daneault J-F, Vergara-Diaz G (2018). Torres ÂFS de OE, Quixadá AP, Fonseca M de L, et al. Complex Upper-Limb Movements Are Generated by Combining Motor Primitives that Scale with the Movement Size. Sci Rep.

[15] Oubre B, Daneault J-F, Jung H-T, Whritenour K, Miranda JGV, Park J (2020). Estimating Upper-Limb Impairment Level in Stroke Survivors Using Wearable Inertial Sensors and a Minimally-Burdensome Motor Task. IEEE Trans Neural Syst Rehabil Eng.

[16] Menegoni F, Milano E, Trotti C, Galli M, Bigoni M, Baudo S (2009). Quantitative evaluation of functional limitation of upper limb movements in subjects affected by ataxia. Eur J Neurol.

[17] Trouillas P, Takayanagi T, Hallett M, Currier RD, Subramony SH, Wessel K (1997). International Cooperative Ataxia Rating Scale for pharmacological assessment of the cerebellar syndrome. The Ataxia Neuropharmacology Committee of the World Federation of Neurology. J Neurol Sci.

[18] Martinez-Manzanera O, Lawerman TF, Blok HJ, Lunsing RJ, Brandsma R, Sival DA (2018). Instrumented finger-to-nose test classification in children with ataxia or developmental coordination disorder and controls. Clin Biomech.

[19] Casamento-Moran A, Yacoubi B, Wilkes BJ, Hess CW, Foote KD, Okun MS, et al. Quantitative Separation of Tremor and Ataxia in Essential Tremor. Ann Neurol. 2020; Available from: 10.1002/ana.25781. 10.1002/ana.25781 PMC8324074 32418250

[20] Fahn S, Elton RL. UPDRS Program Members. Unified Parkinson’s Disease Rating Scale. Recent Development in Parkinson’s Disease, vol. 2: Macmillan; 1987. p. 153–63.

[21] Perez-Lloret S, van de Warrenburg B, Rossi M, Rodríguez-Blázquez C, Zesiewicz T, Saute JAM, et al. Assessment of Ataxia Rating Scales and Cerebellar Functional Tests: Critique and Recommendations. Mov Disord. Wiley Online Library. 2020. 10.1002/mds.28313 33022077

[22] Gagnon C, Mathieu J, Desrosiers J (2004). Standardized finger-nose test validity for coordination assessment in an ataxic disorder. Can J Neurol Sci.

[23] Bouten CV, Koekkoek KT, Verduin M, Kodde R, Janssen JD (1997). A triaxial accelerometer and portable data processing unit for the assessment of daily physical activity. IEEE Trans Biomed Eng.

[24] Jolliffe I, Lovric M (2011). Principal Component Analysis. International Encyclopedia of Statistical Science.

[25] Rasmussen CE, Williams CKI. Gaussian Processes for Machine Learning: MIT Press; 2006.

[26] Han J, Pei J, Kamber M. Data Transformation and Data Discretization. Data Mining: Concepts and Techniques. Elsevier, 2011. p. 111–9.

[27] Leversen JSR, Haga M, Sigmundsson H (2012). From children to adults: motor performance across the life-span. PLoS One.

[28] de Lemos Fonseca M, Daneault J-F, Vergara-Diaz G, Quixadá AP, Souza de Oliveira E Torres AF, Pondé de Sena E (2020). Motor skill acquisition during a balance task as a process of optimization of motor primitives. Eur J Neurosci.

[29] Hoff B (1994). A model of duration in normal and perturbed reaching movement. Biol Cybern.

[30] Koo TK, Li MY (2016). A Guideline of Selecting and Reporting Intraclass Correlation Coefficients for Reliability Research. J Chiropr Med.

[31] Kashyap B, Phan D, Pathirana PN, Horne M, Power L, Szmulewicz D (2020). Objective Assessment of Cerebellar Ataxia: A Comprehensive and Refined Approach. Sci Rep.

[32] Tran H, Pathirana PN, Horne M, Power L, Szmulewicz DJ (2019). Quantitative Evaluation of Cerebellar Ataxia Through Automated Assessment of Upper Limb Movements. IEEE Trans Neural Syst Rehabil Eng.

[33] Maschke M, Gomez CM, Ebner TJ, Konczak J (2004). Hereditary cerebellar ataxia progressively impairs force adaptation during goal-directed arm movements. J Neurophysiol.

